# A Poly(Acrylic Acid)-Based Hydrogel Crosslinked with Hydroxypropylcellulose as a Clarifying Agent in Nickel(II) Solutions

**DOI:** 10.3390/gels11070560

**Published:** 2025-07-21

**Authors:** Rubén Octavio Muñoz-García, Cesar Alexis Ruiz-Casillas, Diego Alberto Lomelí-Rosales, Jorge Alberto Cortés-Ortega, Juan Carlos Sánchez-Díaz, Luis Emilio Cruz-Barba

**Affiliations:** 1 Departamento de Química, Centro Universitario de Ciencias Exactas e Ingenierías, Universidad de Guadalajara, Boulevard Marcelino García Barragán 1421, Guadalajara 44430, Jalisco, Mexico; jorge.cortega@academicos.udg.mx; 2 Licenciatura en Química, Centro Universitario de Ciencias Exactas e Ingenierías, Universidad de Guadalajara, Boulevard Marcelino García Barragán 1421, Guadalajara 44430, Jalisco, Mexico; cesar.ruiz4819@alumnos.udg.mx; 3 Departamento de Ingeniería Química, Centro Universitario de Ciencias Exactas e Ingenierías, Universidad de Guadalajara, Boulevard Marcelino García Barragán 1421, Guadalajara 44430, Jalisco, Mexico; sanchezdiaz@gmail.com (J.C.S.-D.); emilio.cruz@academico.udg.mx (L.E.C.-B.)

**Keywords:** hydroxypropylcellulose, acrylic acid, hydrogels, nickel(II)

## Abstract

Poly(acrylic acid) (PAA) and hydroxypropylcellulose (HPC) hydrogels were synthesized in the absence of a crosslinker. Chemical crosslinking between PAA and HPC was demonstrated through free radical polymerization by a precipitation reaction in acetone as the solvent. These hydrogels exhibited smaller swelling ratios (1 to 5 g H_2_O/g) than homo PAA hydrogels synthesized in water as the solvent. They were swollen in a 0.1 M NaOH solution and subsequently used to remove Ni^2+^ ions from aqueous solutions with concentrations ranging from 1000 to 4000 ppm. The absorption capacity of these hydrogels ranged from 91 to 340 mg of Ni^2+^/g in a rapid 1 h process, and from 122 to 435 mg of Ni^2+^/g in a 24 h process, demonstrating an improvement in Ni^2+^ absorption compared to previously reported hydrogels. The colored 1000 and 2000 ppm Ni^2+^ solutions became clear after treatment, while the PAA-HPC hydrogels turned green due to the uptake of Ni^2+^ ions, which were partially chelated by carboxylate groups as nickel polyacrylate and partially precipitated as Ni(OH)_2_, resulting in an average absorption efficiency of 80%. The hydrogel was able to release the absorbed Ni^2+^ upon immersion in an HCl solution, with an average release percentage of 76.4%, indicating its potential for reuse. These findings support the use of PAA-HPC hydrogels for cleaning Ni^2+^-polluted water. The cost of producing 1 g of these hydrogels in laboratory conditions is approximately 0.2 USD.

## 1. Introduction

Nickel is a transition metal widely used in various industrial processes, including electroplating, mineral processing, stainless steel production, batteries, metal alloys, coins, ceramic coloring, and paints [1,2]. However, nickel contamination poses serious risks to both the environment and human health. Drinking contaminated water, inhaling airborne particles, or consuming contaminated food can lead to harmful effects due to the toxic and carcinogenic properties of nickel [2]. Therefore, it is essential to develop effective and sustainable technologies for the treatment of nickel-polluted water. Hydrogels are polymeric materials capable of absorbing and retaining water, even under pressure, due to their three-dimensional crosslinked structure [3]. This high water-uptake capacity, combined with their functional versatility, makes hydrogels excellent candidates for environmental remediation. The hydrogel’s ability to absorb water makes them suitable for the treatment of heavy metal-contaminated water, due to possible interactions between the functional groups in the polymeric network and the metal ions in solution [4]. Poly(acrylic acid) (PAA)-based hydrogels have been synthesized using various cellulose derivatives. For instance, hydrogels have been prepared with carboxymethyl cellulose (CMC) either without a crosslinker, to function as self-sedimentary adsorbents for cationic basic dyes [5], or with N,N-methylenebis(acrylamide) (NMBA) as a crosslinker, to enhance water retention and nutrient release for plant growth [6]. PAA hydrogels have also been developed using bacterial cellulose nanofibers (BCNFs) and polyethylene glycol diacrylate (PEGDA) as crosslinkers to create scaffolds for vascular and cartilage tissue engineering [7]. Additionally, PAA-HPC hydrogels synthesized with NMBA in aqueous solution have been doped with cesium tungsten bronze, demonstrating their potential in energy-saving smart window applications [8].

The grafting of cellulose with acrylic derivatives has also been achieved through chain transfer polymerization using nanocrystalline cellulose (NCC), poly(N-isopropylacrylamide) (PNIPAM), and PAA [9]. Similarly, microcrystalline cellulose (MCC) has been grafted with PAA and poly(acrylamide) (PAAm) using NMBA as a crosslinker to develop hydrogels capable of absorbing heavy metals such as Cu(II), Pb(II), and Cd(II) from polluted water [10]. Several composite hydrogels based on cellulose or acrylic derivatives have been specifically applied for the removal of Ni^2+^ from aqueous solutions. Crosslinker-free hydrogels include PNIPAM–cellulose acetate (CA) [11], HPC–xanthate [12], and multifunctional systems combining MCC, chitosan (CS), polydopamine (PDA), and polyethyleneimine (PEI) [13]. On the other hand, crosslinked systems have been developed using β-cyclodextrin (βCD) with CMC, MCC, and xylan [14]; D-mannitol xanthate (DMX) with PAA and PAAm [15]; as well as 2-hydroxyethyl acrylate (HEA) and itaconic acid (IA) [16], and βCD with PNIPAM and 2-(dimethylamino)ethyl acrylate (DMAEA) [17]. Among these, the latter two systems achieved the highest Ni^2+^ absorption capacities, reaching 296.7 and 350.8 mg Ni^2+^/g, respectively.

In the present work, we developed PAA–HPC hydrogels via precipitation polymerization without using traditional crosslinkers, aiming to evaluate their performance in the removal of Ni^2+^ from aqueous solutions. The hydrogel containing 5 wt.% HPC exhibited an absorption capacity comparable to the best-performing systems reported in the literature. In contrast, the PAA hydrogel without HPC reached a higher capacity of 435 mg Ni^2+^/g, surpassing previously reported values and demonstrating its potential as a clarifying and purifying agent for Ni^2+^-contaminated water.

## 2. Results and Discussion

### 2.1. Synthesis of the Hydrogels

**Figure 1:**
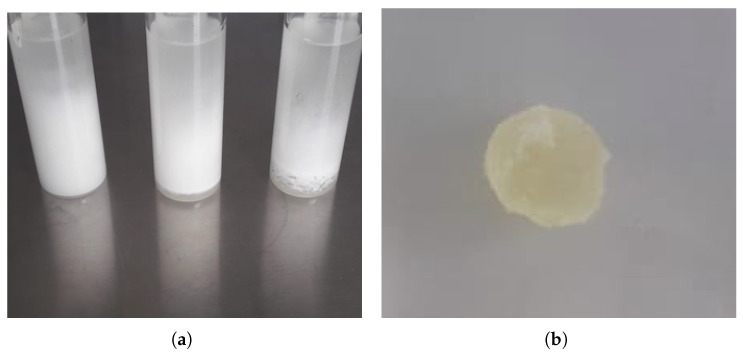
Synthesis. (**a**) Synthesized PAA-HPC hydrogels: 10 wt.% HPC (left), 7.5 wt.% HPC (center), and 5 wt.% HPC (right). (**b**) Dried PAA-HPC xerogel with 20 wt.% HPC.

PAA-HPC hydrogels were synthesized in glass reaction vials using acetone as the solvent, by a free radical polymerization reaction at 55 °C for 12 h. The solids concentration in the system was set to 20 wt.%. For instance, to prepare 2 g of PAA-HPC xerogel with 5 wt.% HPC, the following components were dissolved in 8 g of acetone: 0.1 g HPC (5 wt.% of solids), 1.8812 g acrylic acid (AA), and 0.0188 g benzoyl peroxide (BP) as initiator (1 wt.% relative to AA). Upon completion of the polymerization, the solvent is removed by evaporation, resulting in the formation of the xerogel. Figure 1 shows the polymerized mixture after 12 h and the obtained xerogel after the drying process. The appearance of all the PAA-HPC xerogel formulations is similar. Since the polymerization of AA without a crosslinker yields a hydrogel that is soluble in water, it is not suitable as a control for comparison with the other hydrogel formulations.

Therefore, AA crosslinked with NMBA (1.0 wt.% relative to AA) was used as the control to assess the specific impact of HPC on the hydrogel’s polymeric network. The wt.% yield (Y) was calculated using Equation (1) for each of the two purification steps. Table 1 shows the wt.% yield for the synthesized formulations.

**Figure 2:**
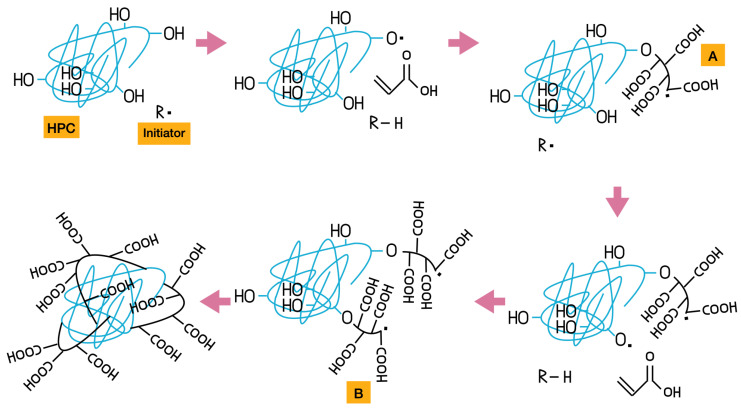
Proposed mechanism for the synthesis of PAA-HPC hydrogels.

The purification steps removed the non-crosslinked polymeric chains, and the average wt.% yield was above 80% for formulations with higher HPC content after drying in an oven at 50 °C. The yield (wt.%) increases as the HPC content increases. At 20 and 25 wt.% HPC, the results show lower variability, which supports the occurrence of chemical crosslinking between AA and HPC. Grafting of cellulose and acrylic derivatives at these concentrations has been reported both with [10,18] and without [19,20] the use of NMBA as a crosslinker. We previously showed that cellulose derivatives can crosslink with acrylic derivatives through a chain transfer radical mechanism in acetone without using conventional crosslinkers like NMBA [11]. When PAAm and CA react in acetone, the reaction proceeds as a precipitation polymerization, in which the polymer formed is insoluble in acetone and gradually precipitates out of the solution as the polymerization progresses [21]. In the present study, PAA and HPC undergo the same type of reaction. Figure 2 shows the proposed mechanism for this reaction. BP, the initiator in this reaction—represented as R· in Figure 2—undergoes homolytic bond cleavage, generating the initial free radical that reacts with both acrylic acid (AA) and hydroxypropylcellulose (HPC) to initiate polymerization. When AA polymerizes, a linear polymer chain is formed. In parallel, when HPC reacts with the initiator, a hydrogen atom from a free hydroxyl group undergoes homolytic cleavage, generating a free radical on the oxygen atom of the cellulose. This radical then reacts with an AA monomer, initiating the formation of a linear AA chain—indicated as the A group in Figure 2—grafted onto the HPC backbone.

Subsequently, a different hydrogen atom from another site on the cellulose undergoes homolytic cleavage, initiating a new AA polymerization process, as represented in compound B. The proposed final step involves the collision of radicals from the growing linear AA chains grafted onto the HPC backbone [10,18,19,20]. This process of grafting polyacrylic acid chains onto HPC through free radical initiation by oxygen is repeated several times until the polymerization reaction is complete.

### 2.2. Swelling

**Figure 3:**
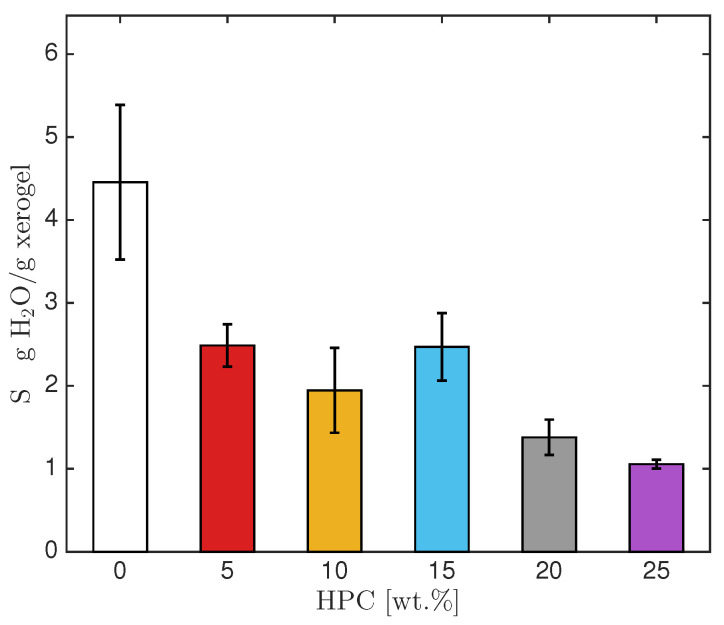
PAA-HPC hydrogels swelling capacity in water.

The swelling capacity of PAA-HPC hydrogels in water and at pH 1 and 13 was calculated using Equation (2). The swelling capacity at pH 1 (in a 0.1 M HCl solution) was nearly the same as in water, averaging around 80% of the value observed in water. However, the swelling capacity at pH 13 (in a 0.1 M NaOH solution) was significantly higher than that in water. At pH 13, the carboxylic acid groups (-COOH) present in the PAA are partially converted into sodium polyacrylate (SPA), which contains highly hydrophilic carboxylate groups (COO^−^) with strong electronegativity and polarity that can physically interact with water molecules. This results in electrostatic repulsion among the carboxylate groups, which expands the polymer chains and leads to greater water uptake [18]. Since the hydrogel’s uptake of the NaOH solution is involved in the Ni^2+^ absorption process, it will be discussed in Section 2.5. Figure 3 shows the swelling capacity of PAA-HPC hydrogels in water. No significant differences were observed in the swelling capacity of formulations containing 5–15 wt.% HPC, nor between those with 20 and 25 wt.% HPC. However, the formulations with the highest HPC content exhibited lower swelling capacities, which was expected since crosslinked hydrogels typically show a decrease in swelling with increased crosslinking density [22]. The formulation with 0 wt.% HPC, containing PAA and 1.0 wt.% NMBA (relative to AA), exhibited a higher swelling capacity than the formulations containing HPC, due to its lower crosslinking density compared to the PAA-HPC hydrogels.

### 2.3. FTIR

**Figure 4:**
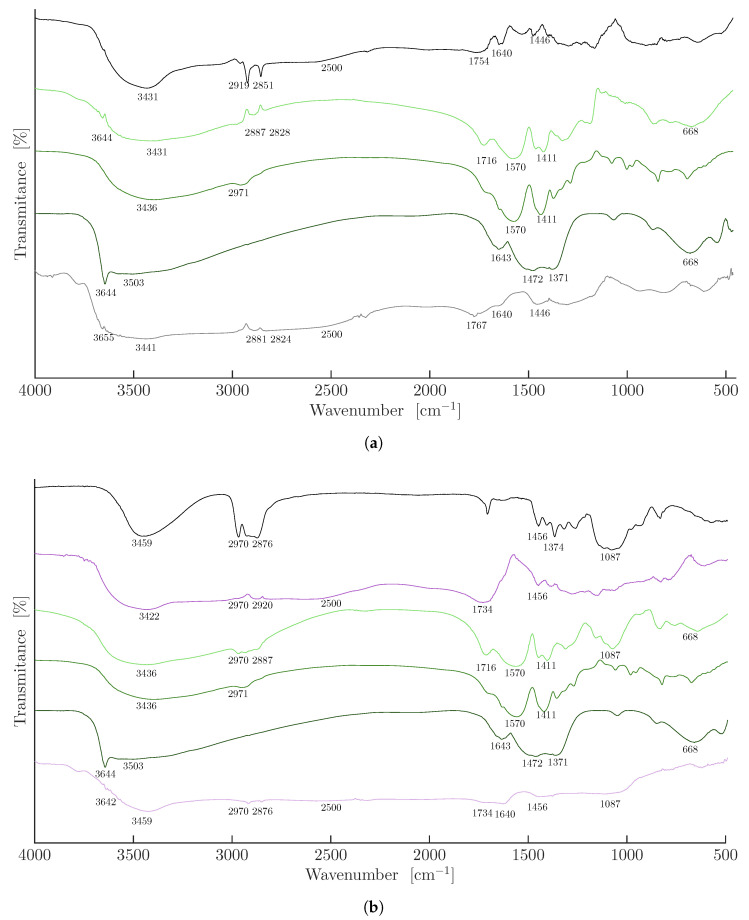
FTIR spectra of PAA-HPC xerogels: (**a**) PAA (0 wt.% HPC) (*black line*), PAA (0 wt.% HPC) with absorbed Ni (*light green line*), pure nickel acrylate (*medium green line*), pure nickel hydroxide (*dark green line*), and PAA (0 wt.% HPC) with absorbed Ni after HCl treatment (*gray line*). (**b**) Pure HPC (*black line*), PAA (25 wt.% HPC) (*purple line*), PAA (25 wt.% HPC) with absorbed Ni (*light green line*), pure nickel acrylate (*medium green line*), pure nickel hydroxide (*dark green line*), and PAA (25 wt.% HPC) with absorbed Ni after HCl treatment (*light purple line*).

After the purification steps, the xerogels were analyzed by FTIR throughout the process, from the initial sample before swelling in NaOH solution to the final sample after Ni^2+^ release induced by HCl addition. These spectra were compared with those of pure materials, including HPC, nickel acrylate, and nickel hydroxide. Figure 4a shows the FTIR spectra of PAA, 0 wt.% HPC (black line). The characteristic bands include broad O-H stretching from 3431 to 2500 cm^−1^ [7,23], and CH_2_ asymmetric and symmetric stretching at 2919 and 2851 cm^−1^, respectively. Additionally, the C=O stretching vibration at 1754 cm^−1^ [24]; the CH_2_ asymmetric bending at 1446 cm^−1^; and a possible absorbed water band at 1640 cm^−1^ [25] were also seen. The characteristic bands of nickel acrylate (medium green line) include strong bands at 1570 and 1411 cm^−1^ corresponding to the asymmetric and symmetric C-O stretching vibrations of the carboxylate group [26,27,28,29], along with the O–H stretching at 3436 cm^−1^ and the C=C stretching at 1639 cm^−1^; the latter signal disappears upon polymerization of AA. For the nickel hydroxide spectrum (dark green line), the characteristic bands include a strong peak at 3644 cm^−1^ corresponding to the non-hydrogen-bonded hydroxyl groups, a broad band at 3436 cm^−1^ attributed to the O-H stretching, a peak at 1371 cm^−1^ typically observed in hydroxide-containing materials, and a broad band at 668 cm^−1^ associated to the Ni-O vibration [30]. When the swollen PAA 0 wt.% HPC hydrogel in 0.1 M NaOH, containing partially deprotonated -COOH groups, absorbs nickel (light green line), a portion of the ions is coordinated by carboxylate sites within the hydrogel matrix, while the remaining ions are precipitated by free hydroxyl groups as Ni(OH)_2_. The observed FTIR bands include a peak at 3644 cm^−1^ corresponding to the non-hydrogen-bonded hydroxyl groups, found in the nickel hydroxide spectrum, strong bands at 1570 and 1411 cm^−1^, corresponding to the asymmetric and symmetric C-O stretching vibrations of the carboxylate group [26,27,28,29], and the broad band at 668 cm^−1^ attributed to the Ni-O vibration [30], along with the -OH and CH_2_ bands present in the PAA 0 wt.% HPC, and a medium C=O peak at 1716 cm^−1^ attributed to partial COOH deprotonation. These results confirm the absorption of nickel(II) both in the form of nickel polyacrylate and nickel hydroxide. Once the hydrogel containing absorbed Ni was placed in HCl solution to release the nickel (gray line), the spectra showed the initial bands of PAA 0 wt.% HPC, along with a band at 3655 cm^−1^ corresponding to the nickel hydroxide, the broad -OH band of PAA ranging from 3441 to 2500 cm^−1^, the reappearance of the C=O peak at 1767 cm^−1^, and a peak attributed to absorbed water at 1640 cm^−1^. These results indicate that traces of both nickel polyacrylate and nickel hydroxide remain even after treatment with HCl. Therefore, the FTIR spectrum of the initial PAA 0 wt.% HPC differs from that of the PAA 0 wt.% HPC after HCl treatment.

Figure 4b shows the FTIR spectra of pure HPC (black line), including a broad -OH stretching vibration at 3459 cm^−1^, CH_2_ asymmetric and symmetric stretching at 2970 and 2876 cm^−1^, respectively, CH_2_ asymmetric and symmetric bending at 1456 and 1374 cm^−1^ [12], and a broad C-O stretching band from 1087 to 1250 cm^−1^ [27]. The PAA-HPC xerogel containing 25 wt.% HPC (purple line) integrates bands of PAA and HPC, such as the broad -OH band ranging from 3441 to 2500 cm^−1^, the strong C=O peak at 1734 cm^−1^, the C-O stretching band from 1087 to 1250 cm^−1^, and the CH_2_ asymmetric bending. When the swollen PAA-HPC hydrogel containing 25 wt.% HPC in 0.1 M NaOH solution absorbs nickel (light green line), a portion of the ions is coordinated by carboxylate sites within the hydrogel matrix, while the remaining ions are precipitated by free hydroxyl groups as Ni(OH)_2_. The observed FTIR bands include a broad -OH band at 3436 cm^−1^, strong bands at 1570 and 1411 cm^−1^, corresponding to the asymmetric and symmetric C-O stretching vibrations of the carboxylate group [26,27,28,29], and the broad band at 668 cm^−1^ attributed to the Ni-O vibration [30]. Also present are the -OH and CH_2_ bands characteristic of PAA, a medium C=O peak at 1716 cm^−1^ attributed to partial COOH deprotonation, and a broad C-O stretching band ranging from 1087 to 1250 cm^−1^. The 3644 cm^−1^ peak corresponding to the non-hydrogen-bonded hydroxyl groups, typically observed in the nickel hydroxide spectrum (dark green line), was not detected, likely due to the saturation of the -OH signal caused by the excess hydroxyl groups present in HPC. These results confirm the absorption of nickel(II) both in the form of nickel polyacrylate and nickel hydroxide, as well as the crosslinking of HPC with PAA. Once the hydrogel containing absorbed Ni was placed in HCl solution to release the nickel, the spectra showed the initial bands of PAA, along with a band at 3642 cm^−1^ corresponding to the nickel hydroxide, the broad -OH band of PAA ranging from 3459 to 2500 cm^−1^, the reappearance of the C=O peak at 1734 cm^−1^, and a peak attributed to absorbed water at 1640 cm^−1^, as well as the bands at 1456 and 1087 cm^−1^ present in the HPC spectrum. The appearance of the 3642 cm^−1^ signal is likely due to a reduction in –OH signal saturation caused by HPC hydrolysis in 0.1 M HCl solution. These results indicate that traces of both nickel polyacrylate and nickel hydroxide remain even after treatment with HCl. Therefore, the FTIR spectrum of the initial 25 wt.% HPC xerogel (purple line) differs from that of the 25 wt.% HPC xerogel after HCl treatment (light purple line).

### 2.4. Microstructural Observation

**Figure 5:**
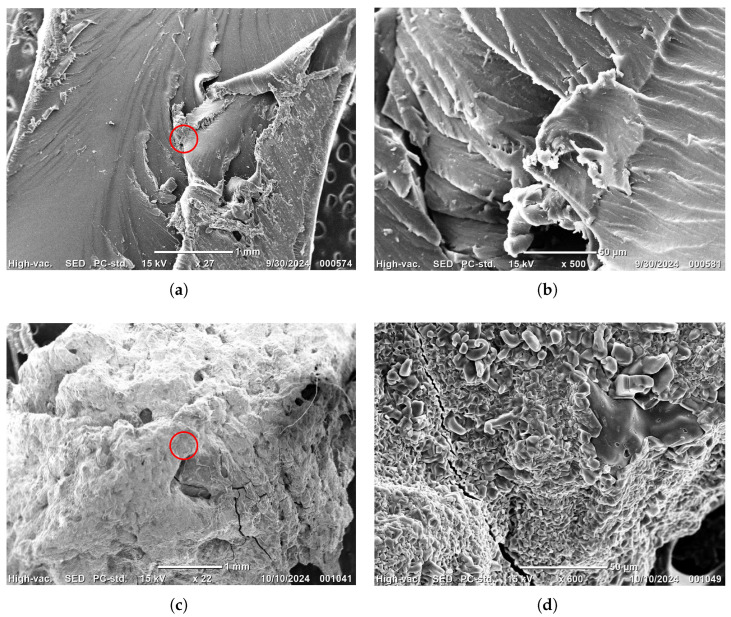
Scanning electron microscopy micrographs of PAA-HPC xerogels: (**a**) 5 wt.% HPC; (**b**) close-up of the red-circled region in image (**a**); (**c**) 5 wt.% HPC with adsorbed Ni^2+^ suggesting nickel polyacrylate and Ni(OH)_2_ crystals; (**d**) close-up of the red-circled region in image (**c**).

Formulations of PAA-HPC xerogels were analyzed by scanning electron microscopy (SEM) before and after Ni^2+^ absorption. Figure 5a shows an image of a purified sample of 5 wt.% HPC xerogel. While some areas of the surface are smooth, others exhibit cavities and protrusions, suggesting microstructure variations. Figure 5b presents a close-up of the red-circled region in Figure 5a, revealing a cracked surface that indicates the formation of fissures during the drying process. Figure 5c shows an image of the 5 wt.% HPC xerogel after the absorption process, where the xerogel was completely encapsulated, likely in the form of nickel polyacrylate and Ni(OH)_2_ crystals. These crystals formed in the hydrogel when Ni^2+^ ions penetrated the hydrogel loaded with hydroxyl ions, leading to precipitation and chelation with carboxylates. Figure 5d presents a close-up of the red-circled region in Figure 5c, revealing that small crystals—apparently in the form of nickel polyacrylate and Ni(OH)_2_—were embedded in the cavities and on the cracked surface, as confirmed by the FTIR discussion in Section 2.3. Similar crystals were obtained and visualized by SEM micrographs using the same methodology with PNIPAM-CA hydrogels [11]. In the following step, these crystals are removed from the hydrogel using a 0.1 M HCl solution. Subsequently, the hydrogel is dried at room temperature for reuse.

### 2.5. Nickel(II) Absorption

**Figure 6:**
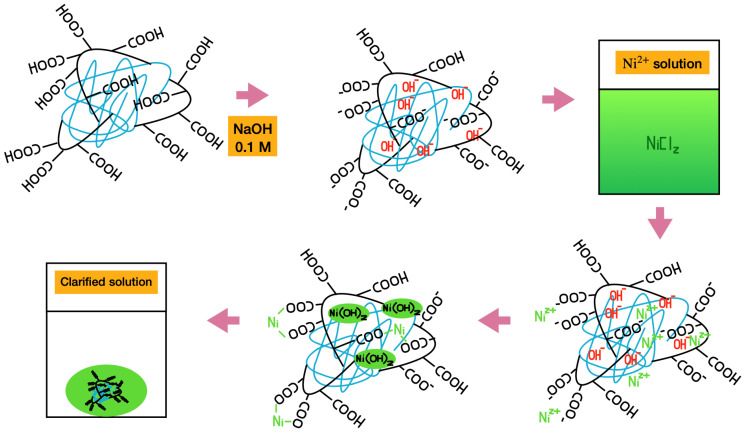
Nickel(II) solution clarification process using PAA-HPC xerogels (*spectator ions are present in the system but were omitted to provide a clearer view of the figure*).

**Figure 7:**
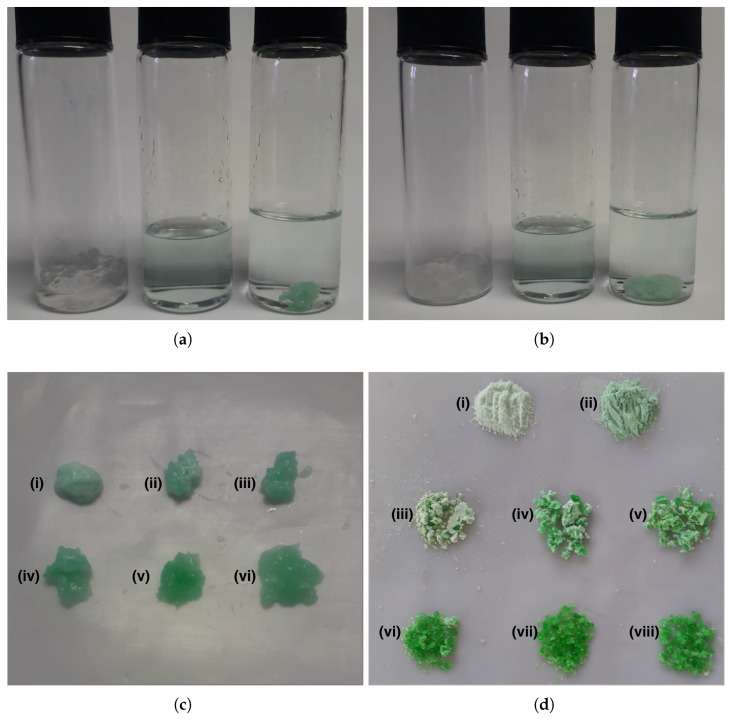
Absorption process of nickel(II) by PAA–HPC hydrogels over a one-hour period. (**a**) Filtered 5 wt.% HPC hydrogel swollen in 0.1 M NaOH solution (**left vial**), 2000 ppm Ni^2+^ solution (**center vial**), and 5 wt.% HPC hydrogel with absorbed Ni and clarified solution (**right vial**). (**b**) Filtered 0 wt.% HPC hydrogel swollen in 0.1 M NaOH solution (**left vial**), 2000 ppm Ni^2+^ solution (**center vial**), and 0 wt.% HPC hydrogel with absorbed Ni and clarified solution (**right vial**). (**c**) Hydrogels with absorbed Ni at 0–25 wt.% HPC in samples (**i**–**vi**), respectively. (**d**) (**i**) Pure nickel acrylate, (**ii**) pure nickel hydroxide, and dried xerogels with absorbed Ni at 0–25 wt.% HPC in samples (**iii**–**viii**), respectively.

Figure 6 shows a schematic representation of the Ni absorption process by PAA-HPC hydrogels. Purified PAA-HPC xerogels were swollen in 0.1 M NaOH solution. The final weight of the hydrogel depends on both the HPC content (wt.%) and the immersion time in the NaOH solution: the weight increases with longer immersion times but decreases with higher HPC contents. During swelling, the hydrogel absorbs hydroxyl ions with the solution, leading to the partial deprotonation of carboxylic acid groups (-COOH) and the formation of carboxylate groups (−COO^−^). Subsequently, the swollen hydrogels were immersed in a 2000 ppm Ni^2+^ solution at a mass ratio of 3:1 relative to the hydrogel. After a rapid 1 h process, Ni^2+^ ions penetrate the hydrogel, which turns green. Two distinct processes occur during Ni^2+^ penetration. First, the hydrogel collapses, resulting in a reduction of its initial mass, attributed to the chelation of absorbed Ni^2+^ by carboxylate groups (−COO^−^) [31]. Second, Ni(OH)_2_ precipitated due to the presence of free hydroxyl ions within the hydrogel network [11,26]. Since the PAA is in a crosslinked solid phase, the neutralization reaction with the –COOH groups is not expected to reach 100% yield. Eventually, the initially green-colored solution became clear. Figure 7a shows the Ni absorption process for the PAA–HPC hydrogel containing 5 wt.% of HPC. The filtered swollen hydrogel in NaOH solution (left vial) was introduced into a 2000 ppm Ni^2+^ solution at a mass ratio of 3:1 relative to the hydrogel (center vial). After a 1 h process, the hydrogel collapsed and turned green while the solution became clear. The apparent volume of the solution increased due to the collapse of the hydrogel and the release of absorbed water (right vial).

Figure 7b shows the same process as in Figure 7a, applied to the 0 wt.% HPC composition. In both cases, the decrease in hydrogel mass due to collapse and the corresponding increase in solution volume are evident. A similar behavior was observed for the rest of the formulations. As shown in Figure 7c, the filtered PAA–HPC hydrogels after Ni absorption range from 0 wt.% HPC in sample (i) to 25 wt.% HPC in sample (vi), with 5 wt.% intervals between samples. The green color observed in the hydrogels is attributed to the presence of nickel acrylate (light green) and nickel hydroxide (medium green). Pure samples of these compounds are shown in Figure 7d as samples (i) and (ii), respectively. From Figure 7c, it can be observed that the intensity of the green color increases with the HPC content. This suggests that at 0 wt.% HPC, the absorbed Ni^2+^ is predominantly present as nickel polyacrylate, while at higher HPC contents, Ni^2+^ is preferentially absorbed in the form of Ni(OH)_2_. Figure 7d shows the dried PAA–HPC xerogels with absorbed Ni, ranging from 0 wt.% HPC in sample (iii) to 25 wt.% HPC in sample (viii), with 5 wt.% intervals between samples. This figure displays the same color pattern as Figure 7c and further supports the interpretation. This behavior could be attributed to the crosslinking density of the PAA–HPC hydrogels, suggesting that as the HPC content increases, the polymeric network becomes tighter, hindering the neutralization of –COOH groups by hydroxyl ions and leading to the precipitation of nickel through interaction with free hydroxyl groups. After the Ni absorption process, the hydrogels with absorbed Ni^2+^ were removed from the solution to spectrophotometrically quantify the concentration of Ni^2+^ remaining in the solution and calculate the amount of nickel adsorbed using Equations (3) and (4). The Ni^2+^ concentration of the remaining solution after treatment with PAA-HPC hydrogels was quantified using a calibration curve (0–2000 ppm) at 396 nm with a Unico S-2150UV UV/Visible spectrophotometer.

Table 2 presents key data on the Ni absorption process, including the concentrations of Ni^2+^ in the remaining solutions, the Ni^2+^ absorption capacities, and the percentage of Ni^2+^ removal. After a rapid 1 h treatment with the PAA-HPC hydrogel, the Ni^2+^ concentration in the remaining solution decreases, on average, to approximately one-third of its initial value, corresponding to an average Ni removal of approximately two-thirds, regardless of the HPC wt.%. Extending the treatment time to 24 h further reduces the Ni^2+^ concentration to about one-fifth of the initial value, resulting in an average Ni removal of 80% across all hydrogel compositions. HPC wt.% has no significant impact on the Ni^2+^ concentration or the Ni wt.% removed from the solution after treatment with the PAA-HPC hydrogel. All hydrogel compositions exhibit a similar ratio of Ni^2+^ absorbed per gram of NaOH. For any composition, this ratio increases as the treatment time extends from 1 to 24 h, since most of the Ni^2+^ is absorbed within the first hour, while the remaining NaOH and carboxylates continue to react, forming additional nickel polyacrylate and Ni(OH)_2_ over the subsequent 23 h.

The 0.1 M NaOH swelling capacity values for PAA-HPC xerogels containing 0–25 wt.% HPC were 90, 55, 36, 35, 28, and 22 g of NaOH solution per xerogel gram, respectively. The values shown in Table 2 were converted to pure NaOH absorption capacity. Since the 0 wt.% HPC hydrogel exhibited a higher swelling capacity, using 1 g of each swollen hydrogel in NaOH solution resulted in the 0 wt.% HPC sample containing less xerogel mass than the one with 25 wt.% HPC. As a result, when the Ni^2+^ absorption capacity was calculated per gram of xerogel—by dividing the amount of Ni absorbed (in milligrams) by the xerogel mass—a higher value was obtained for the 0 wt.% HPC sample.

For instance, the difference in Ni^2+^ absorption capacity per gram of xerogel between the hydrogel with 0 wt.% HPC $\left(340\;\frac{\mathrm{mg}\;{\mathrm{Ni}}^{2+}}{g\;\mathrm{xerogel}}\right)$ and that with 25 wt.% HPC $\left(91\;\frac{\mathrm{mg}\;{\mathrm{Ni}}^{2+}}{g\;\mathrm{xerogel}}\right)$ showed in Table 2—despite both removing nearly the same percentage of Ni—can be attributed to their differing NaOH swelling capacities. The Ni^2+^ absorption capacity values shown in Table 2 increase from a range of 91–340 to 122–435 mg Ni^2+^/g when the treatment time is extended from 1 to 24 h.

A stoichiometric overview of the 1 h Ni absorption process, based on Table 2, indicates that 1 g of 0 wt.% HPC xerogel contains 0.0136 mol of –COOH groups. The NaOH swelling capacity for this composition is 0.364 g of NaOH per gram of xerogel, corresponding to 0.0091 mol of NaOH. As a result, the –COOH groups are only partially converted to −COO^−^. It is expected that some hydroxide ions remain unreacted, as they may be unable to reach the –COOH groups due to the crosslinked structure of the polymeric network in the solid phase. The amount of Ni^2+^ absorbed is 340 mg, corresponding to 0.00579 moles. It is therefore assumed that a portion of the divalent Ni is chelated by the −COO^−^ groups, while the remaining Ni^2+^ is precipitated by unreacted hydroxide ions. Based on Figure 7d, sample (iii) exhibits a predominantly light green color, which corresponds to nickel acrylate as observed in sample (i). In contrast, 1 g of 25 wt.% HPC xerogel contains 0.0103 mol of –COOH groups. The NaOH swelling capacity for this composition is 0.089 g of NaOH per gram of xerogel, corresponding to 0.0022 mol of NaOH. As a result, the –COOH groups are only partially converted to −COO^−^. It is expected that some hydroxide ions remain unreacted, as they may be unable to reach the –COOH groups due to the highly crosslinked structure of the polymeric network in the solid phase. The amount of Ni^2+^ absorbed is 91 mg, corresponding to 0.00155 moles. It is therefore assumed that a portion of the divalent Ni is chelated by the −COO^−^ groups, while the remaining Ni^2+^ is precipitated by unreacted hydroxide ions. Based on Figure 7d, sample (viii) exhibits a predominantly medium green color, which corresponds to nickel hydroxide as observed in sample (ii). These results suggest that at higher HPC contents, the absorption of Ni^2+^ occurs predominantly in the form of Ni(OH)_2_, whereas at 0 wt.% HPC, it primarily occurs in the form of nickel polyacrylate.

The 0 and 5 wt.% HPC hydrogel formulations achieved the highest nickel absorption capacities and were immersed in solutions containing 1000, 3000, and 4000 ppm of Ni^2+^ for 1 h to compare their effectiveness at both lower and higher Ni^2+^ concentrations. Table 3 shows that the Ni absorption capacity was similar across all concentrations. However, the highest removal efficiency was observed for the 1000 ppm solution, while the lowest was for the 4000 ppm solution. These results were expected, since the 4000 ppm solution contained the highest amount of Ni. In addition, the 1000 and 2000 ppm solutions became clear after treatment, while the others showed a reduction in color intensity but did not become clear. When the hydrogel-to-Ni solution ratio was increased to 1:10 or 1:30, the solutions did not become clear due of the high Ni^2+^ mass content. The absorption capacity is limited by the availability of −COO^−^ and hydroxyl groups present in the swollen hydrogel.

**Figure 8:**
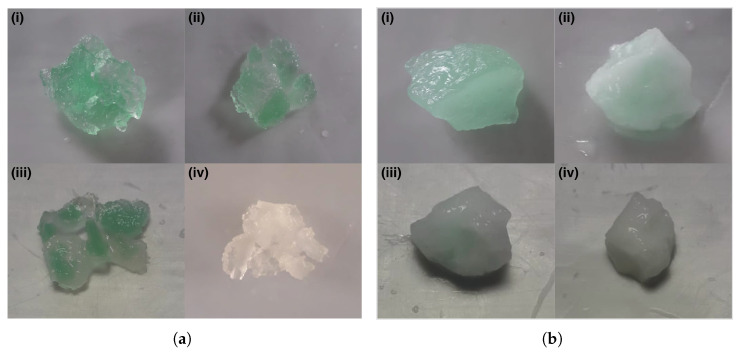
Nickel ions release from PAA-HPC hydrogels with absorbed Ni in HCl solution. (**a**) 5 wt.% HPC. (**b**) 0 wt.% HPC.

Figure 8 illustrates the Ni release process in 5 and 0 wt.% HPC hydrogel compositions containing absorbed Ni, immersed in 0.1 M HCl solution. For both panels a and b, image (i) corresponds to the hydrogel before immersion, while image (iv) shows the hydrogel after the release process is complete. Images (ii) and (iii) reveal that Ni release begins at the surface and progresses toward the center of the hydrogel, providing evidence of Ni^2+^ penetration throughout the material.

A close examination of image (i) in Figure 8a,b reveals that the green color intensity is greater for the hydrogel containing 5 wt.% HPC content than the one with 0 wt.% HPC. This suggests a higher amount of Ni absorbed, likely in the form of Ni(OH)_2_ for the 5 wt.% HPC composition. The Ni release (wt.%) for hydrogels containing 0–25 wt.% HPC was 62.1, 70.4, 88.7, 82.4, 69.8, and 85.0 wt.%, respectively, as calculated using Equation (5). These results suggest that the release of chelated Ni is more difficult than the release of Ni from absorbed Ni(OH)_2_.

Once the release process was completed, the hydrogels were washed with distilled water, dried, and reused. The Ni^2+^ absorption capacities after one day, for formulations containing 0–25 wt.% HPC, were 213.1, 115.3, 93.4, 75.2, 60.9, and 55.7 mg Ni^2+^/g, respectively. These values were lower than those reported in Table 2 for the first use, suggesting that the presence of unreleased Ni^2+^ reduces the hydrogel’s absorption capacity, likely due to coordination between −COO^−^ groups and retained Ni^2+^ ions and undissolved Ni(OH)_2_.

Our work represents an improvement in the Ni^2+^ absorption capacity of hydrogels for removing Ni^2+^ from aqueous solutions compared to previously reported cellulose-based, acrylic-based, and other hydrogel systems summarized in Table 4.

Although the highest absorption values achieved are obtained with 0 wt.% HPC composition (e.g., PAA-NMBA), hydrogel formulations containing 5 wt.% HPC or higher exhibit a greater polymerization reaction yield, as shown in Table 1, as well as a higher Ni-releasing percentage for reuse, compared to the 0 wt.% HPC formulation. Moreover, cellulose and acrylic derivatives are relatively inexpensive materials and offer potential for exploring novel free-radical polymerization reactions without the use of traditional crosslinkers.

## 3. Conclusions

The yield and swelling capacity of PAA-HPC xerogels depend on the HPC content: increasing HPC improves the polymerization yield but reduces water and NaOH solution swelling, indicating crosslinking with PAA.

PAA–HPC hydrogels acted as clarifying agents for 2000 ppm Ni^2+^ solutions, significantly improving their transparency after treatment. In addition, they effectively removed nickel from aqueous media, achieving up to 80% removal after 24 h. The corresponding absorption capacities reached 435 and 263 mg Ni^2+^/g for the 0 and 5 wt.% HPC formulations, respectively.

Ni^2+^ uptake occurs via chelation and precipitation mechanisms, as confirmed by FTIR and SEM. Recovery of absorbed Ni^2+^ is feasible using HCl, with an average efficiency of 76%.

These findings highlight the potential of PAA-HPC hydrogels as efficient materials for removing Ni^2+^ from contaminated water.

## 4. Materials and Methods

### 4.1. Materials

AA 99%, HPC 99% (80,000 Da), NMBA 99%, BP 99%, nickel(II) chloride 99%, sodium hydroxide 97%, hydrochloric acid (37%, ACS reagent), and acetone (ACS grade) were purchased from Sigma-Aldrich (St. Louis, MO, USA) and used as received. Double-distilled water was used.

### 4.2. Synthesis of the Hydrogels

PAA-HPC hydrogels were synthesized in glass reaction vials, using acetone as the solvent. The total solid concentration in the system was set at 20 wt.%, with the HPC content varying from 0 to 25 wt.% relative to the total solids in 5 wt.% intervals. The remaining portion consisted of AA, with BP used as an initiator at 1 wt.% relative to AA. The materials were dissolved in acetone, which represented 80 wt.% of the total mixture, and polymerized at 55 °C for 12 h. Afterwards, the PAA-HPC hydrogels were extracted from the reaction vial and dried in an oven at 50 °C to obtain the PAA-HPC xerogels. The xerogels were subjected to a purification step to remove residual unreacted compounds. As a purification step, xerogels were weighed and immersed in an excess of distilled water for 3 days to allow for the diffusion of soluble impurities. Subsequently, the swollen hydrogels were weighed and dried in an oven at 50 °C.

This purification cycle was repeated twice to ensure adequate removal of residuals prior to further characterization. The wt.% reaction yield (Y) was calculated using

(1) $$Y=\frac{w_{f}}{w_{0}}\times 100$$

where $w_{f}$ and $w_{0}$ represent the sample’s weight at the end of the purification step and its initial weight, respectively. All the experiments were performed three times and the standard deviation was calculated. Since the polymerization of AA without a crosslinker yields a hydrogel that is soluble in water, it is not suitable as a control group for comparison with the other hydrogel formulations. Therefore, AA crosslinked with NMBA was used as the control group.

### 4.3. Swelling

Xerogel samples were immersed in an excess of water at room temperature (25 ± 1 °C) for 3 days during which water was replaced every other day. The swelling capacity (S) was calculated using

(2) $$S=\frac{w_{t}-w_{0}}{w_{0}}$$

where $w_{t}$ and $w_{0}$ represent the sample’s weight at maximum swelling value and its dry weight, respectively. All the experiments were performed three times and the standard deviation was calculated.

### 4.4. Nickel(II) Absorption

The xerogels were immersed in a 0.1 M NaOH solution and allowed to swell for 3 days, using a xerogel-to-NaOH solution ratio of 1:100. Then, the swollen hydrogels were placed in a 2000 ppm Ni^2+^ solution. In each case, the hydrogel-to-Ni solution ratio was set at 1:3. After 1 and 24 h, the hydrogel and the solution were weighed, and the absorbance of the remaining solution was measured at 396 nm using an S-2150UV UV/Visible Spectrophotometer (UNICO, Dayton, NJ, USA) to determine the amount of Ni^2+^ that was not absorbed by the hydrogel. The Ni^2+^ concentration of the remaining solution was quantified using a calibration curve (0–2000 ppm). For each Ni(II) treatment solution, a calibration curve was prepared, ranging from 0 to its corresponding concentration. The amount of absorbed Ni^2+^ by the hydrogel was calculated using a material balance

(3) $${\mathrm{Ni}}_{a}^{2+}={\mathrm{Ni}}_{0}^{2+}-{\mathrm{Ni}}_{r}^{2+}$$

where ${\mathrm{Ni}}_{a}^{2+}$, ${\mathrm{Ni}}_{0}^{2+}$, and ${\mathrm{Ni}}_{r}^{2+}$ represent the absorbed amount, the initial amount, and the remaining amount of nickel in solution at room temperature, respectively. For each experiment, the wt.% of nickel absorbed (i.e., removed from the solution) was calculated using

(4) $$\mathrm{wt}.\%\;{\mathrm{Ni}}_{a}^{2+}=\left(\frac{\mathrm{wt}.\;{\mathrm{Ni}}_{a}^{2+}}{\mathrm{wt}.\;{\mathrm{Ni}}_{0}^{2+}}\right)\times 100$$

where $\mathrm{wt}.\;{\mathrm{Ni}}_{a}^{2+}$ represents the amount of nickel absorbed, and $\mathrm{wt}.\;{\mathrm{Ni}}_{0}^{2+}$ represents the amount of Ni^2+^ present in the solution added to the hydrogel at room temperature. All the experiments were performed three times and the standard deviation was calculated. To demonstrate the hydrogel’s reusability, the hydrogel with absorbed Ni^2+^ was dried, yielding the green solids shown in Figure 7d. Then, the xerogels were immersed in 0.1 M HCl solution. The xerogel-to-HCl solution ratio was set at 1:100 to partially dissolve the nickel hydroxide and partially exchange the chelated ${\mathrm{Ni}}^{2+}$ ions with $H^{+}$, thereby regenerating the –COOH groups. The amount of Ni released ($\mathrm{wt}.\%,\;{\mathrm{Ni}}_{r}^{2+}$) was quantified spectrophotometrically using a calibration curve, and the release percentage was calculated using Equation (5).

(5) $$\mathrm{wt}.\%\;{\mathrm{Ni}}_{r}^{2+}=\left(\frac{\mathrm{wt}.\;{\mathrm{Ni}}_{H}^{2+}}{\mathrm{wt}.\;{\mathrm{Ni}}_{a}^{2+}}\right)\times 100$$

where $\mathrm{wt}.\;{\mathrm{Ni}}_{a}^{2+}$ represents the amount of absorbed nickel, and $\mathrm{wt}.\;{\mathrm{Ni}}_{H}^{2+}$ represents the amount of ${\mathrm{Ni}}^{2+}$ present in the HCl solution after treatment at room temperature. Once the hydrogel became clear, it was dried at room temperature to initiate a new ${\mathrm{Ni}}^{2+}$ absorption cycle.

### 4.5. FTIR

The xerogel samples were prepared in 1 mm-thick KBr pellets, obtained by compression molding (1.0–2.0 mg of xerogel with dried KBr to complete a total of 80 mg for the pellet). The FTIR spectra between 4000 and 500 ${\mathrm{cm}}^{-1}$ were recorded using a Spectrum One FTIR spectrophotometer (Perkin Elmer, Waltham, MA, USA). All spectra were obtained at ambient temperature with a resolution of 4 ${\mathrm{cm}}^{-1}$, and 50 scans were carried out for each sample. The final spectra of xerogels were normalized.

### 4.6. Microstructural Observation

The morphology analysis was carried out using a model JCM-6000PLUS scanning electron microscope (SEM) (JEOL Ltd., Tokyo, Japan) operated at 15 kV. The samples were gold-coated before the analysis. 

## Figures and Tables

**Table 1:** Yield of the synthesis of PAA-HPC xerogels.

Composition	1st Purif.	2nd Purif.
wt.% HPC	Step (%)	Step (%)
0	51.6 ± 2.8	84.3 ± 0.7
5	70.2 ± 10.9	90.9 ± 3.9
10	84.5 ± 5.0	95.3 ± 2.1
15	85.9 ± 4.4	95.4 ± 1.5
20	86.8 ± 0.3	98.1 ± 0.7
25	86.6 ± 1.1	97.8 ± 0.5

**Table 2:** Nickel (II) concentrations remaining and absorption capacities after treatment with PAA-HPC hydrogel in 2000 ppm Ni(II) solutions.

HPC	Swelling	After 1 h				After 24 h			
**(wt.%)**	** $\frac{g\;\mathrm{NaOH}}{g\;\mathrm{xerogel}}$ **	**[Ni^2+^] (ppm)**	** $\frac{\mathrm{mg}\;{\mathrm{Ni}}^{2+}}{g\;\mathrm{xerogel}}$ **	** $\frac{\mathrm{mg}\;{\mathrm{Ni}}^{2+}}{g\;\mathrm{NaOH}}$ **	**Ni % Removed**	**[Ni^2+^] (ppm)**	** $\frac{\mathrm{mg}\;{\mathrm{Ni}}^{2+}}{g\;\mathrm{xerogel}}$ **	** $\frac{\mathrm{mg}\;{\mathrm{Ni}}^{2+}}{g\;\mathrm{NaOH}}$ **	**Ni % Removed**
0 ^1^	0.364	631 ± 87	340 ± 87	934	62 ± 4	446 ± 34	435 ± 12	1194	74 ± 2
5	0.220	638 ± 117	207 ± 20	941	63 ± 6	364 ± 50	263 ± 9	1198	80 ± 3
10	0.144	592 ± 112	153 ± 5	1059	69 ± 7	417 ± 46	185 ± 5	1280	79 ± 2
15	0.139	708 ± 25	126 ± 16	908	59 ± 2	228 ± 40	202 ± 6	1451	86 ± 3
20	0.112	751 ± 127	107 ± 15	952	62 ± 4	322 ± 58	161 ± 5	1438	84 ± 3
25	0.089	686 ± 170	91 ± 13	1025	66 ± 9	225 ± 44	122 ± 3.4	1373	88 ± 3

^1^ This formulation was crosslinked with NMBA.

**Table 3:** Ni absorption of PAA-HPC hydrogels immersed in 3000 and 4000 ppm of nickel(II) solutions.

	1000 ppm Ni^2+^			3000 ppm Ni^2+^			4000 ppm Ni^2+^		
**wt.% HPC**	**[Ni^2+^] (ppm)**	** $\frac{\mathrm{mg}\;{\mathrm{Ni}}^{2+}}{g}$ **	**Removed Ni (%)**	**[Ni^2+^] (ppm)**	** $\frac{\mathrm{mg}\;{\mathrm{Ni}}^{2+}}{g}$ **	**Removed Ni (%)**	**[Ni^2+^] (ppm)**	** $\frac{\mathrm{mg}\;{\mathrm{Ni}}^{2+}}{g}$ **	**Removed Ni (%)**
0	118	206	87	1361	302	42	2069	331	35
5	129	104	75	1583	135	34	2088	187	35

**Table 4:** Reported studies on the removal of nickel(II) from aqueous solutions.

Composite	Single Use	Reference	Year
Hydrogel	Absorption		
βCD-CMC	17.29 mg Ni^2+^/g	[14]	2019
PNIPAM-CA	38.4 mg Ni^2+^/g	[11]	2025
HPC-xanthate	114.3 mg Ni^2+^/g	[12]	2016
HEA-IA	225.4 mg Ni^2+^/g	[16]	2021
CS-PDA-PEI-MCC	261.8 mg Ni^2+^/g	[13]	2022
PAA-HPC	263 mg Ni^2+^/g	present work	2025
DMX-PAAm-PAA	296.7 mg Ni^2+^/g	[15]	2024
βCD-PNIPAM-DMAEA	350.8 mg Ni^2+^/g	[17]	2025
PAA-NMBA	435 mg Ni^2+^/g	present work	2025

## Data Availability

The original contributions presented in this study are included in the article. Further inquiries can be directed to the corresponding author.
